# Supplementary material for: Deep representation features from DreamDIAXMBD improve the analysis of data-independent acquisition proteomics
Source: Commun Biol. 2021 Oct 14;4:1190. doi: 10.1038/s42003-021-02726-6 (PMC8517002; doi:10.1038/s42003-021-02726-6)
Supplement: Supplementary file 2 — Supplementary Information [file 42003_2021_2726_MOESM2_ESM.pdf]

Supplementary Information

Deep representation features from DreamDIA^{XMBD} improve the analysis of data-independent acquisition proteomics

Mingxuan Gao[1,2], Wenxian Yang[3], Chenxin Li[1], Yuqing Chang[1], Yachen Liu[1,2], Qingzu He[2,4], Chuan-Qi Zhong[5], Jianwei Shuai[2,4], Rongshan Yu[1,2,3,*] and Jiahuai Han[2,5,6,*]

* Corresponding author. Email: rsyu@xmu.edu.cn, jhan@xmu.edu.cn

[1] School of Informatics, Xiamen University, Xiamen, China.
[2] National Institute for Data Science in Health and Medicine, Xiamen University, Xiamen, China.
[3] Aginome Scientific, Xiamen, China.
[4] College of Physical Science and Technology, Xiamen University, Xiamen, China.
[5] State Key Laboratory of Cellular Stress Biology, School of Life Science, Xiamen University, Xiamen, China.
[6] Research Unit of Cellular Stress of CAMS, School of Medicine, Xiamen University, Xiamen, China.

Supplementary Figures

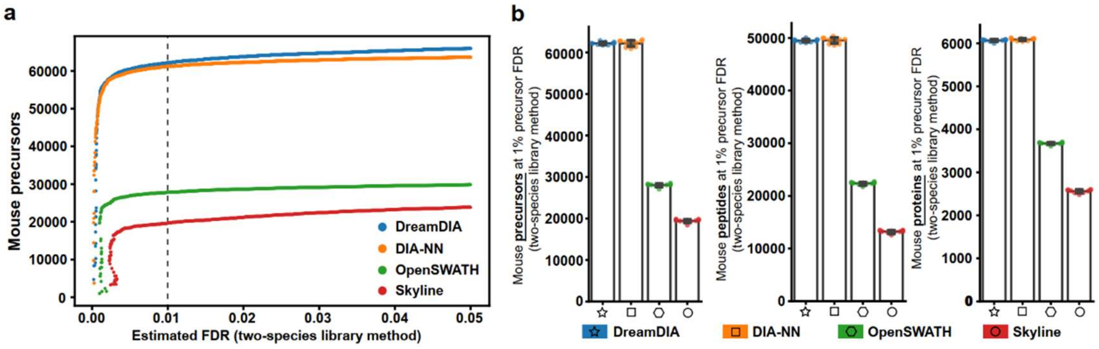

Supplementary Figure 1. Identification performance evaluation of DreamDIA on the MCB dataset with two-species library method. The spectral library built from DDA master samples was used as sample-specific library for analysis. (a) Identification performance on the S1-1 run of the MCB dataset. The numbers of mouse precursors identified at different FDRs were plotted. Each point stands for an *Arabidopsis* (false positive) precursor and its discriminant score as a cut-off value. The x-axis value stands for the estimated FDR, calculated as the number of *Arabidopsis* precursor with higher discriminant score than this cut-off value divided by the number of all the precursors with higher discriminant score than this cut-off value. The y-axis value stands for the number of mouse precursors with higher discriminant score than this cut-off value. (b) Identification performance on all 10 samples of the MCB dataset. The numbers of mouse precursors, peptides and proteins at 1% precursor FDR (the respective numbers indicated by the dashed line in (a)) were plotted. Each error bar stands for the mean and standard deviation of the results of n = 10 biologically independent runs.

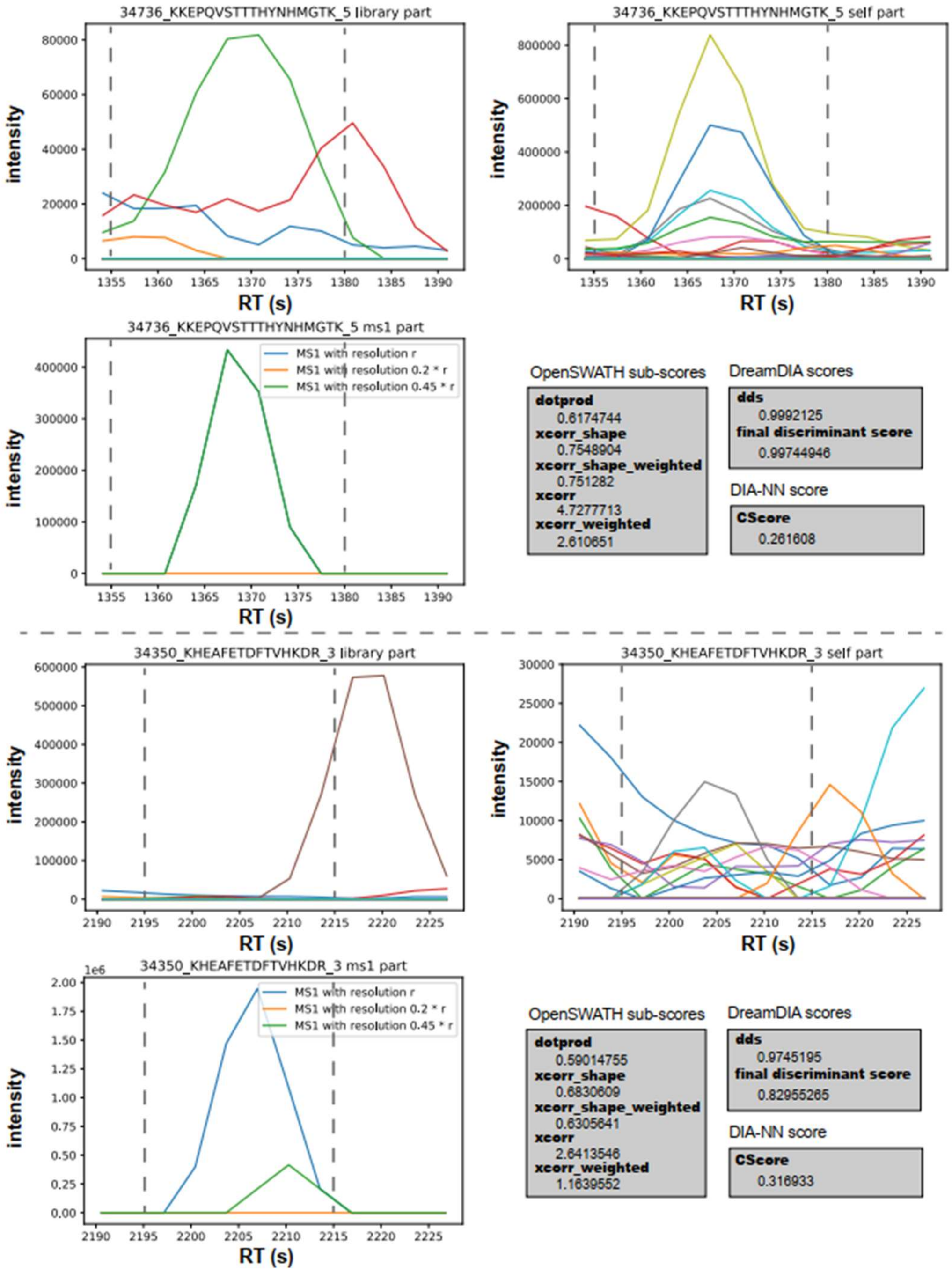

Supplementary Figure 2. RSM examples of the identified mouse precursors by DreamDIA while missed by DIA-NN in the S1-1 run of the MCB dataset. *Library* part, *self* part and *ms1* part of the RSMs are plotted. The dashed lines indicate the elution profile ranges determined by the MS1 XICs. Elution-related sub-scores provided by OpenSWATH and discriminant scores provided by DIA-NN and DreamDIA are also listed. *r*, the basic resolution than can be specified by users according to the acquisition resolution. *dds*, deep discriminant score, which is calculated by the deep representation model in DreamDIA for each precursor in the spectral library to indicate its probability of belonging to a real peptide.

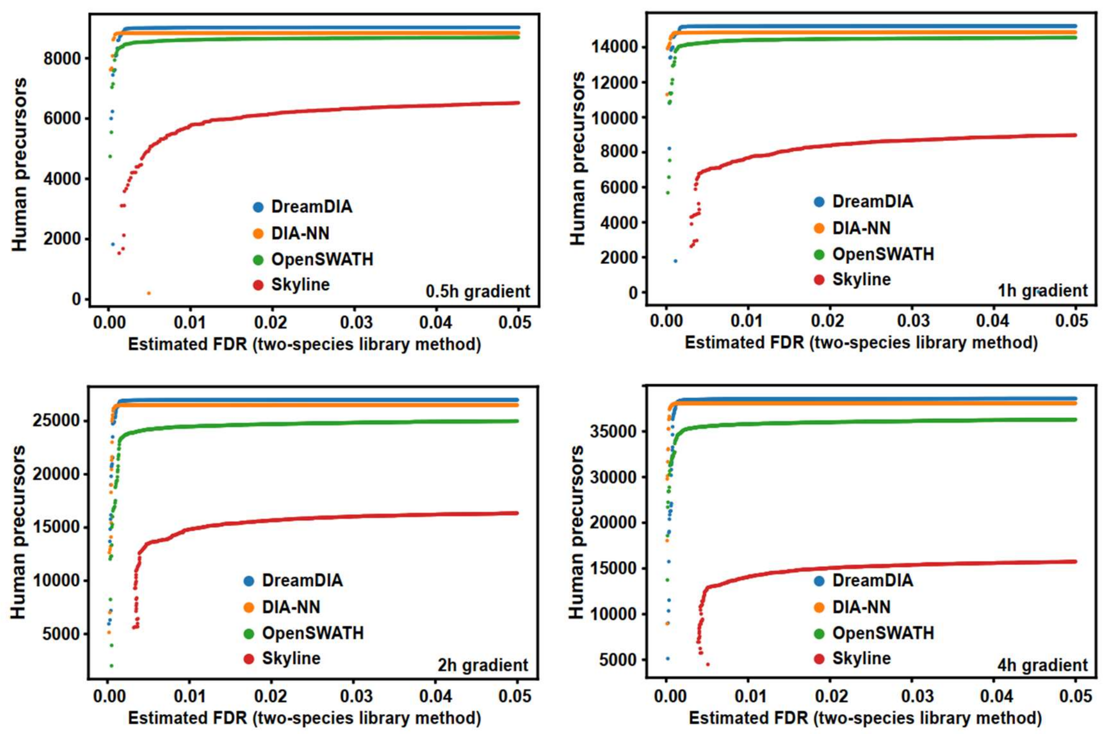

Supplementary Figure 3. Benchmarking of identification performance of DreamDIA on the HeLa dataset (acquired on QExactive HF, Thermo Fisher Scientific) used in the DIA-NN paper. Different gradient lengths from 0.5h to 4h were tested. For each run, the sample-specific library was built by DIA-Umpire and equivalent *Arabidopsis* precursors were spiked into the library as false positive targets for FDR estimation. The numbers of human precursors identified at different FDRs were plotted.

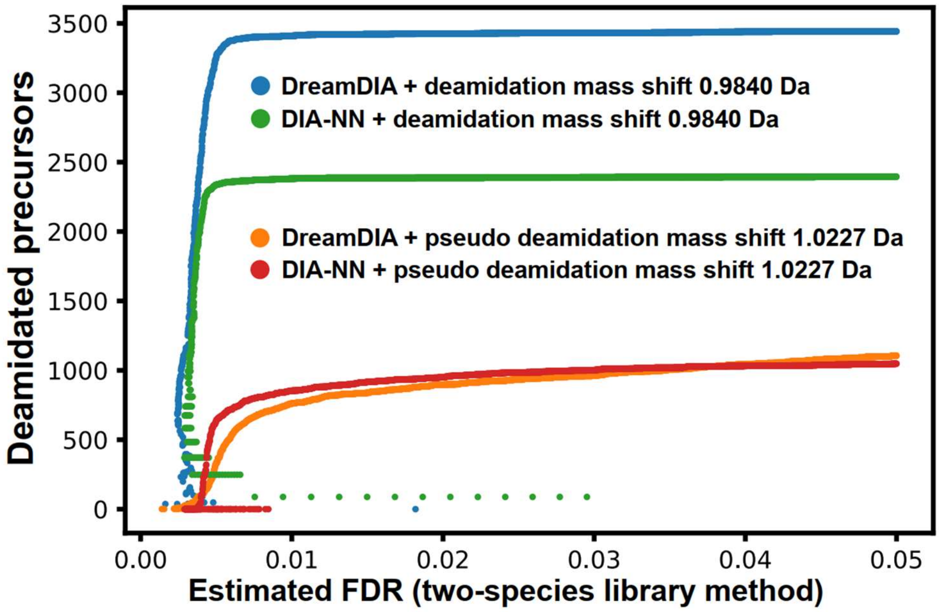

Supplementary Figure 4. Evaluation of deamidated peptide identification performance of DreamDIA. The S1-1 run of the MCB dataset was analyzed twice by DreamDIA and DIA-NN respectively, first using the library containing common deamidated peptides with mass shift of 0.9840 Da, and then using the library containing pseudo-deamidated peptides with mass shift of 1.0227 Da. The numbers of deamidated precursors identified at different FDRs estimated by the two-species library method were plotted.

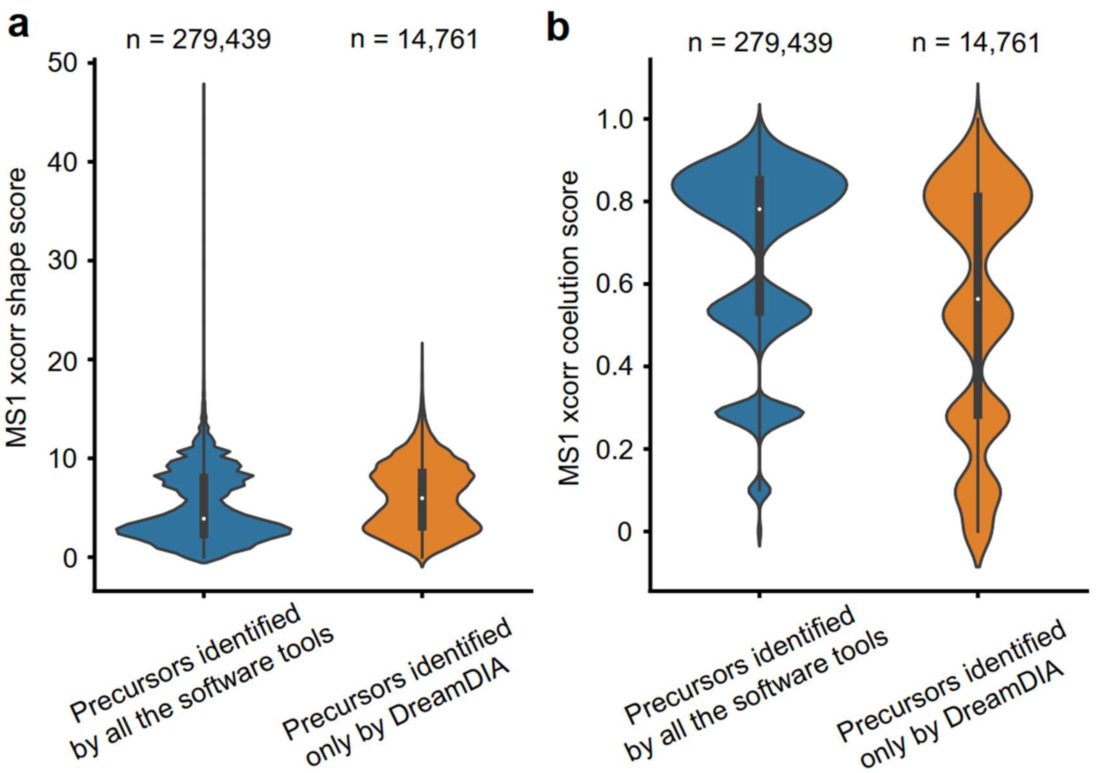

Supplementary Figure 5. Distributions of two MS1-related sub-scores provided by OpenSWATH, (a) MS1 xcorr shape score, (b) MS1 xcorr coelution score in all 10 runs of the MCB dataset. The blue violins indicate the intersection of precursors identified by all the four software tools at 1% proxy precursor FDR, and the orange violins indicate precursors identified exclusively by DreamDIA at 1% proxy precursor FDR. All the sub-scores were obtained from the reports of OpenSWATH without FDR control.

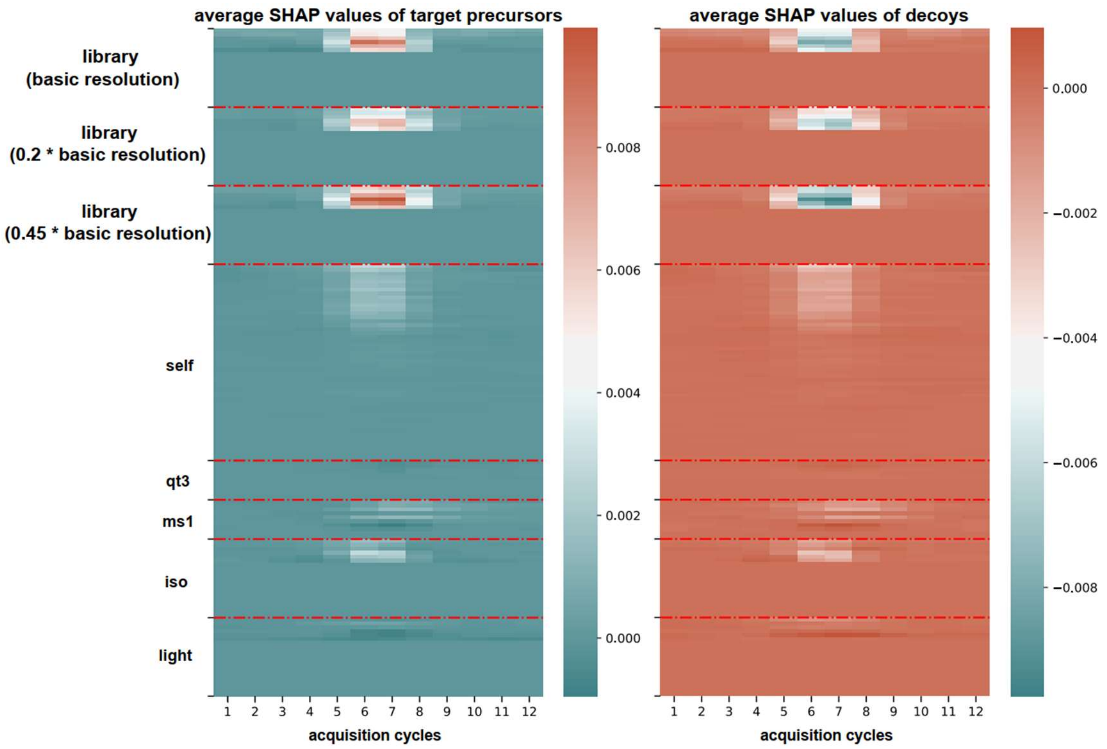

Supplementary Figure 6. Average SHAP values of randomly picked 10000 RSMs (5402 target precursors and 4598 decoys) from the training set. Higher SHAP values indicate higher feature importance in the RSM.

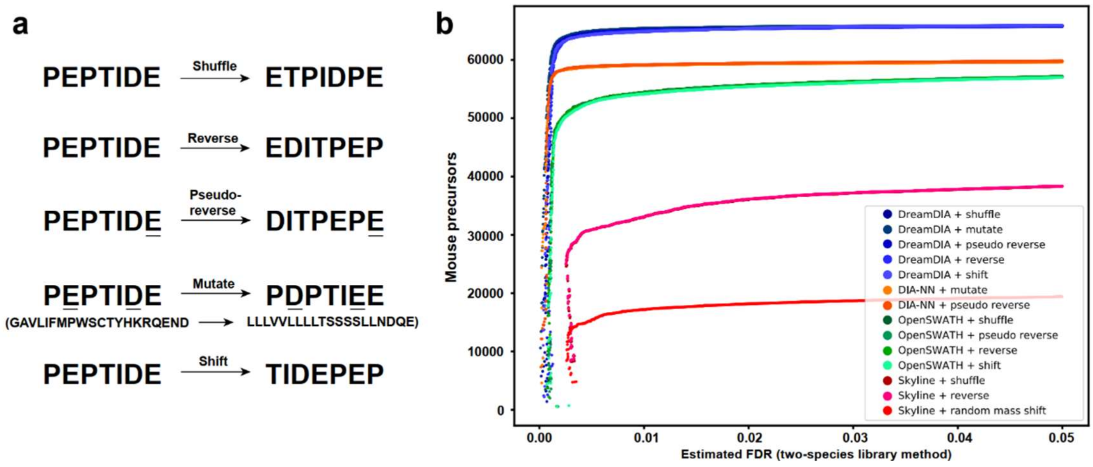

Supplementary Figure 7. Evaluation of the decoy generation methods. (a) Five decoy generation methods integrated in DreamDIA. For DreamDIA, OpenSWATH and Skyline, the *shuffle* algorithm is used as the default method, while DIA-NN used the *mutate* algorithm by default. (b) The influence of decoy generation methods to the precursor identification performance evaluated on the S1-1 run of the MCB dataset. All the decoy generation methods that are compatible for each software tool were tested.

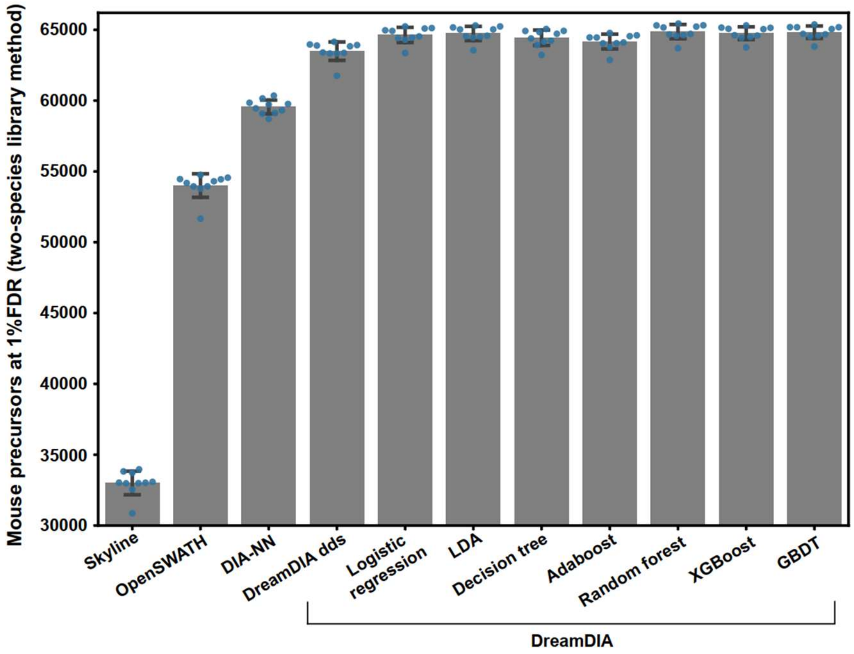

Supplementary Figure 8. Comparison of identification performance of various discriminative models on the MCB datasets. The numbers of mouse precursors at 1% precursor FDR by the two-species library method were plotted. Each error bar stands for the mean and standard deviation of the results of n = 10 biologically independent runs.

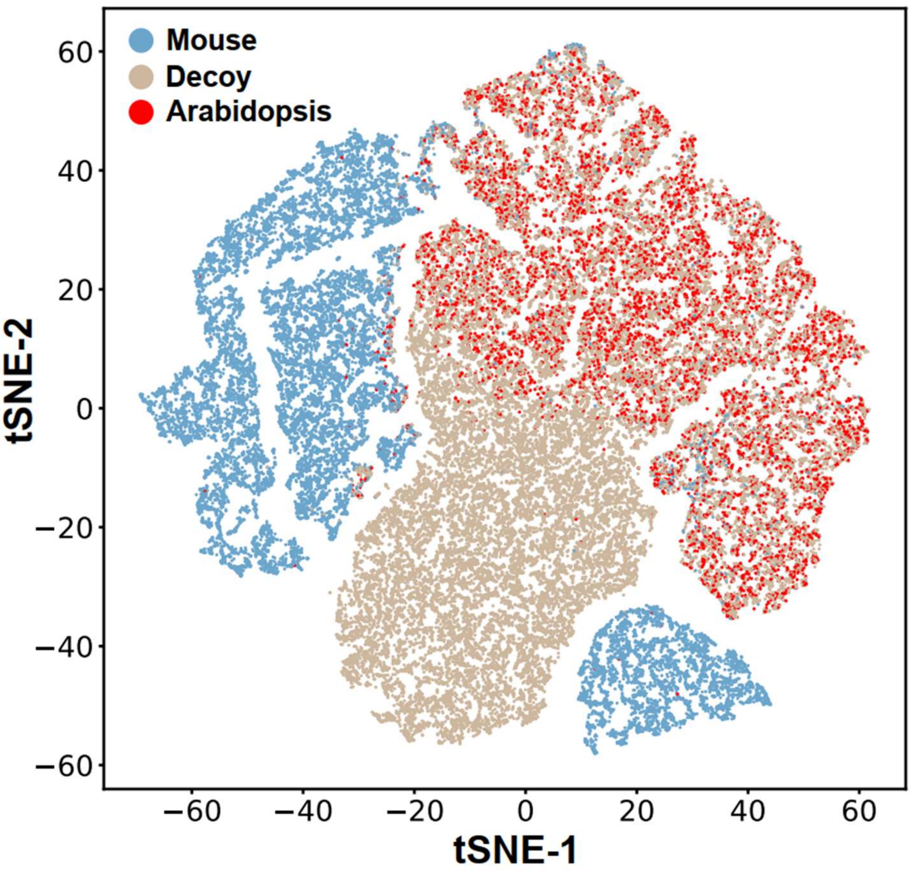

Supplementary Figure 9. tSNE of the extracted 16-dimension deep representation features by DreamDIA from the S1-1 run of the MCB dataset. Each point stands for a candidate RSM of a precursor before the final discrimination. Only 5% of the extracted RSMs are displayed here for better visualization (mouse: 16887; *Arabidopsis*: 7927; decoy: 34018).

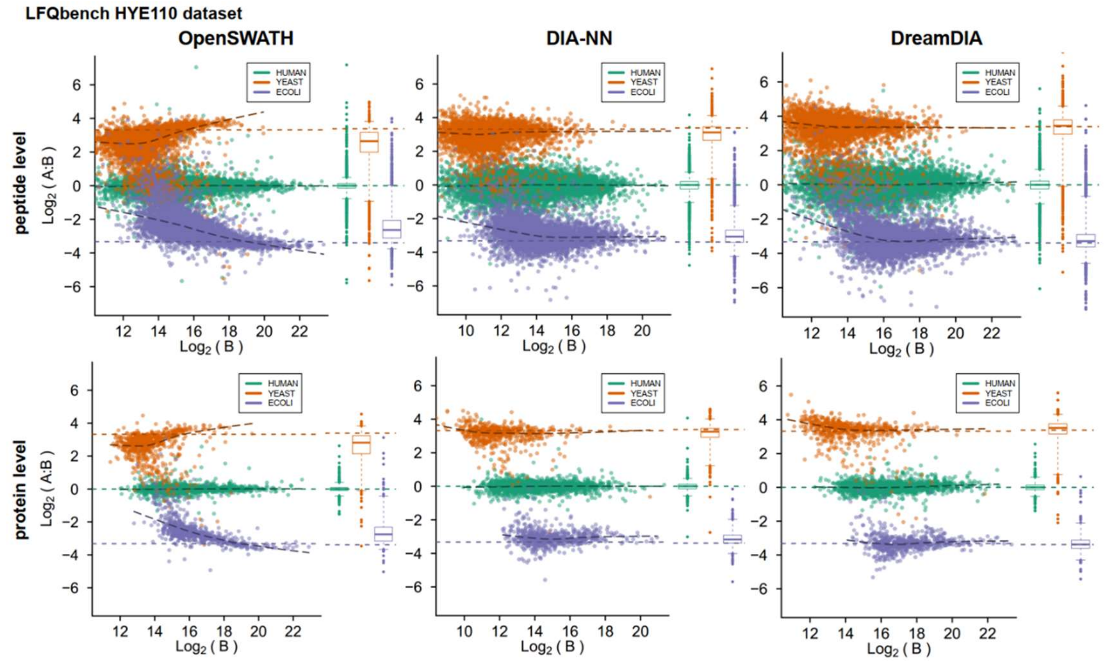

Supplementary Figure 10. Quantification performance evaluation with LFQbench HYE110 dataset. In this dataset, peptides from three species (human, yeast and *E.coli*) were mixed for sample preparation to obtain two groups of samples containing known peptide concentration ratios (A_{human}:B_{human} = 1:1, A_{yeast}:B_{yeast} = 10:1 and $A_{E.coli}$:$B_{E.coli}$ = 1:10, three parallel injections for each group), which are indicated by the colored dashed lines. Peptides (the first row) and proteins (the second row) identified at 1% precursor FDR reported by the software tools themselves were retained, and the calculated ratios were plotted. Boxplot elements: center line, median; boxes, interquartile range; whiskers, percentiles 1-99; points, outliers.

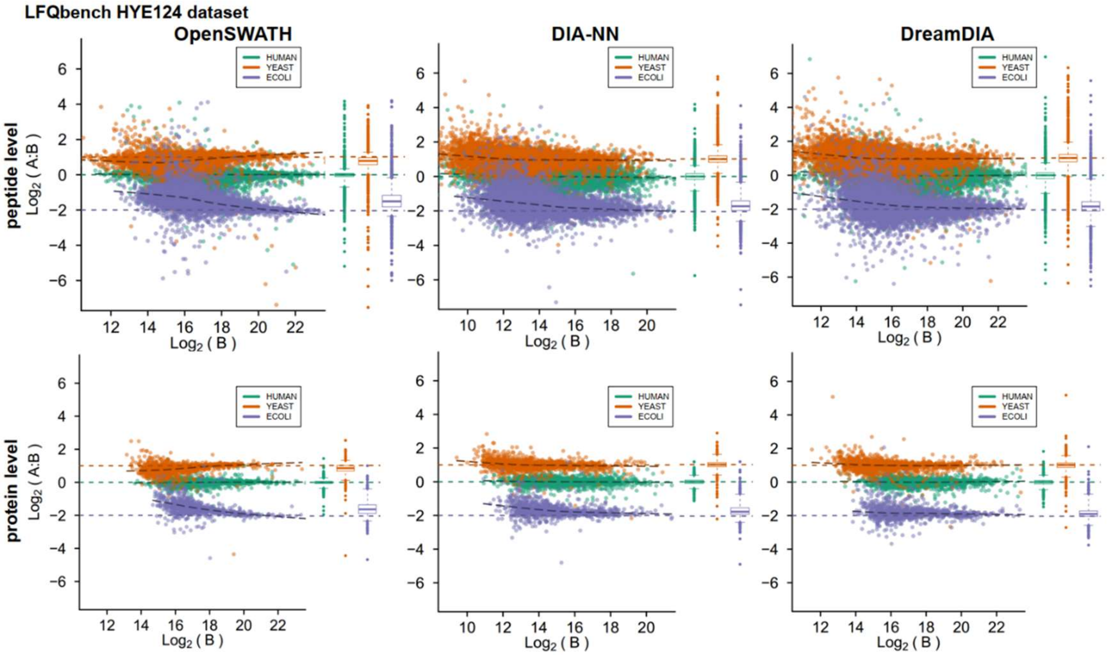

Supplementary Figure 11. Quantification performance evaluation with LFQbench HYE124 dataset. In this dataset, peptides from three species (human, yeast and *E.coli*) were mixed for sample preparation to obtain two groups of samples containing known peptide concentration ratios (A_{human}:B_{human} = 1:1, A_{yeast}:B_{yeast} = 2:1 and $A_{E.coli}$:$B_{E.coli}$ = 1:4, three parallel injections for each group), which are indicated by the colored dashed lines. Peptides (the first row) and proteins (the second row) identified at 1% FDR reported by the software tools themselves were retained, and the calculated ratios were plotted. Boxplot elements: center line, median; boxes, interquartile range; whiskers, percentiles 1-99; points, outliers.

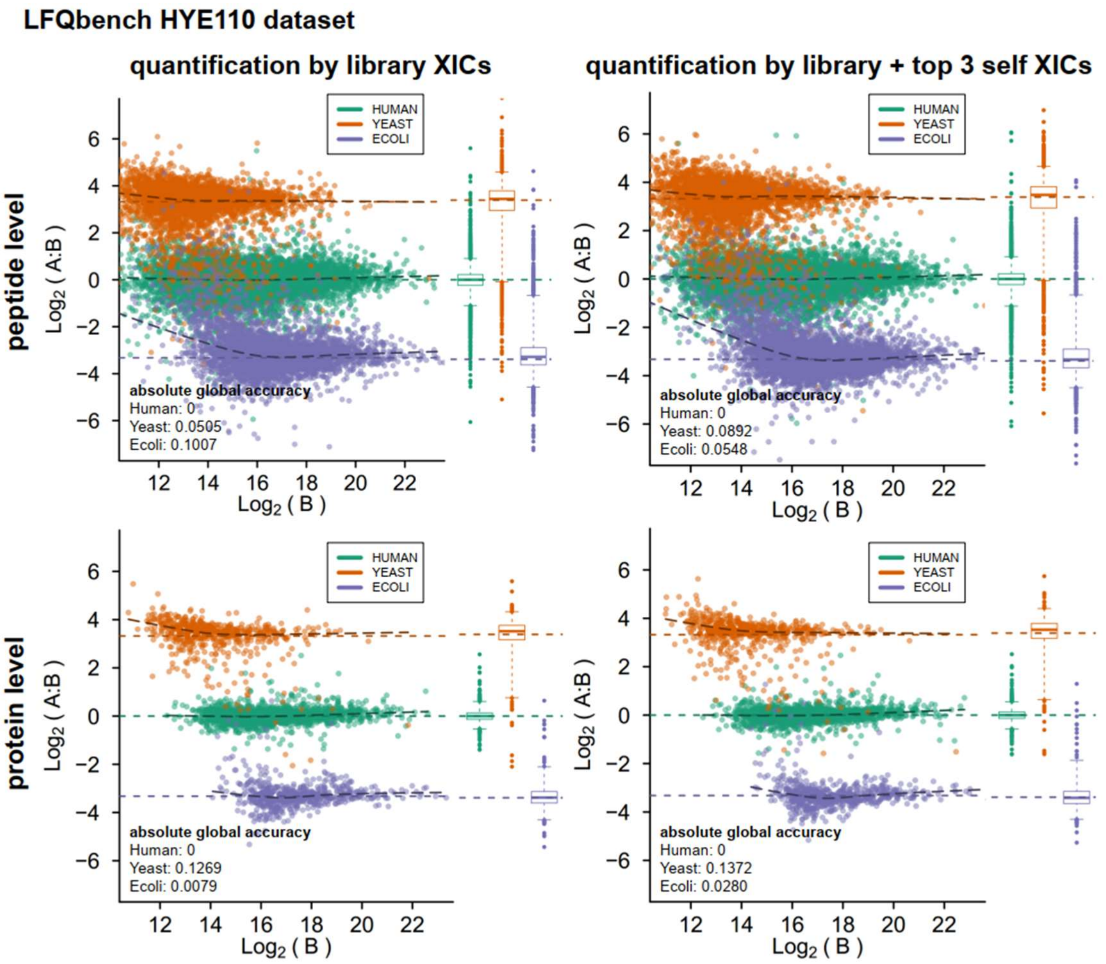

Supplementary Figure 12. Evaluation of the quantification performance of DreamDIA on the LFQbench HYE110 dataset when more fragment ions are included for computation. The global accuracy metric provided by LFQbench software suite reflects the median deviation of calculated log-ratios to the expected values. The absolute value of the global accuracy is displayed here for more intuitive comparison. More results are shown in Supplementary Table S3. Boxplot elements: center line, median; boxes, interquartile range; whiskers, percentiles 1-99; points, outliers.

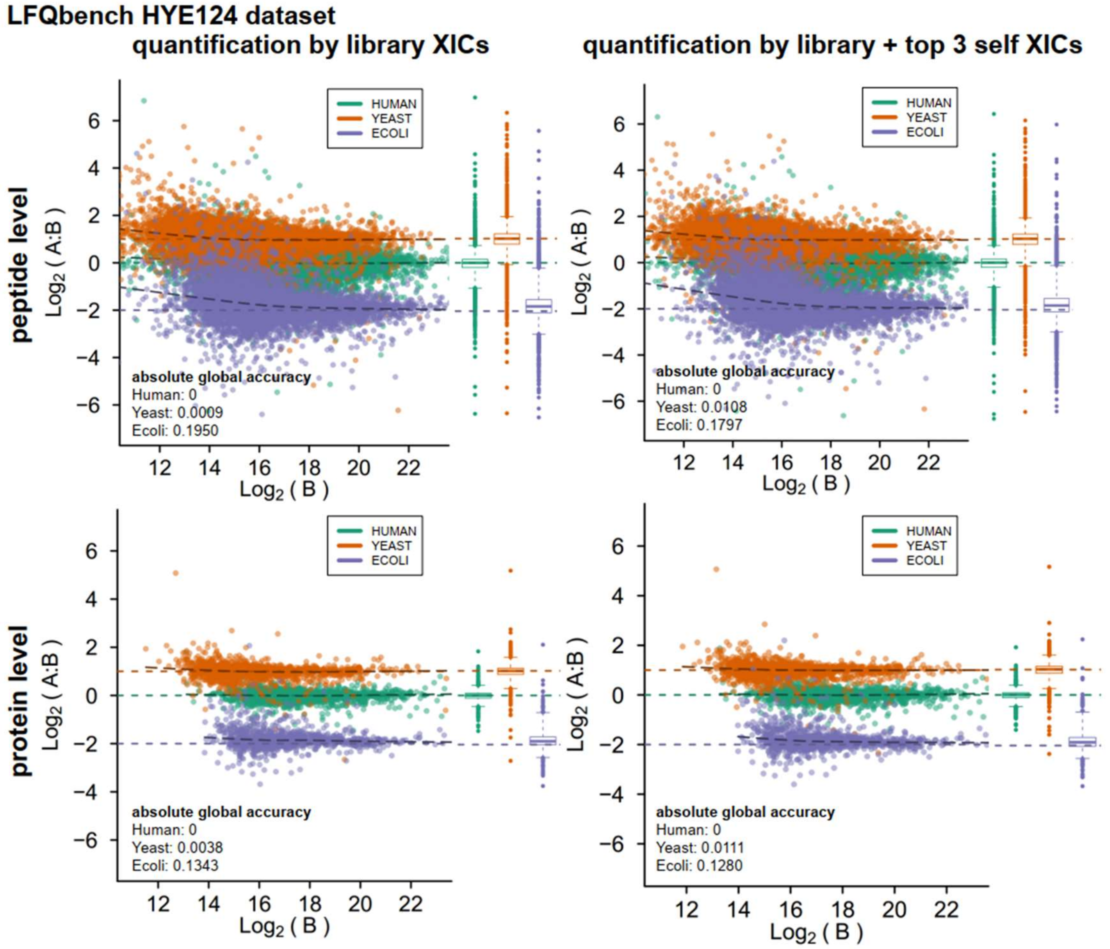

Supplementary Figure 13. Evaluation of the quantification performance of DreamDIA on the LFQbench HYE124 dataset when more fragment ions are included for computation. The global accuracy metric provided by LFQbench reflects the median deviation of calculated log-ratios to the expected values. The absolute value of the global accuracy is displayed here for more intuitive comparison. Boxplot elements: center line, median; boxes, interquartile range; whiskers, percentiles 1-99; points, outliers.

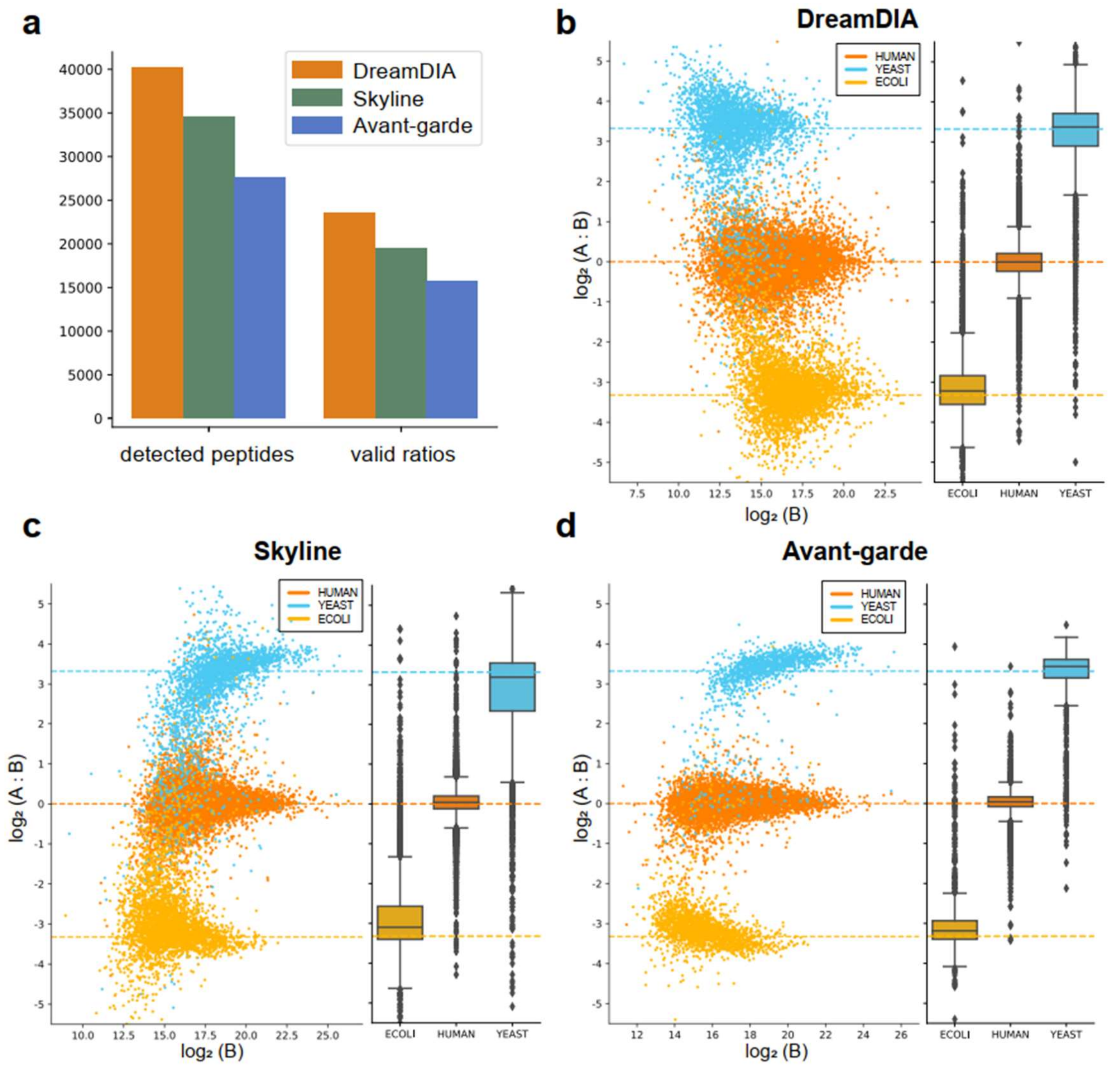

Supplementary Figure 14. Benchmarking of DreamDIA with Avant-garde on the LFQbench HYE110 dataset. (a) Number of peptides identified, and valid peptide quantification ratios reported by LFQbench. Peptides at 1% FDR reported by the software tools themselves were retained, and the calculated ratios of (b) DreamDIA, (c) Skyline and (d) Avant-garde were plotted. Boxplot elements: center line, median; boxes, interquartile range; whiskers, 1.5x interquartile range; points, outliers.

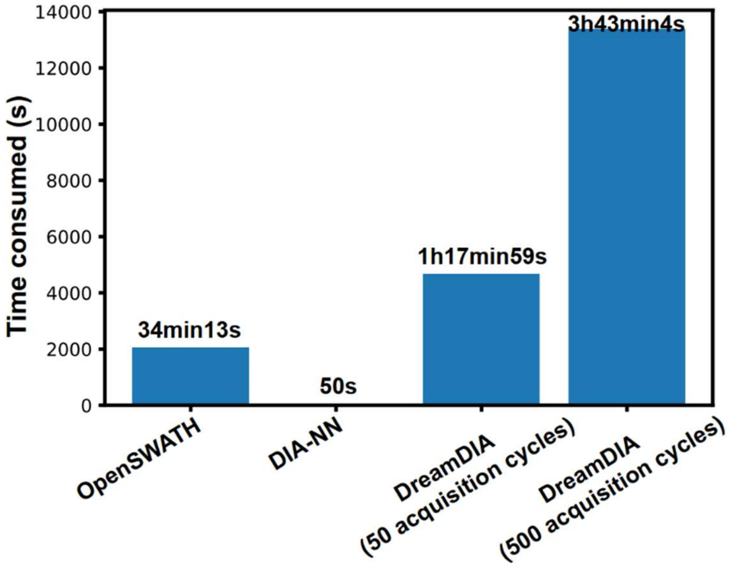

Supplementary Figure 15. Computational efficiency evaluation of DreamDIA. The S1-1 run of the MCB dataset with the two-species library containing 158, 226 precursors in total was processed by OpenSWATH, DIA-NN and DreamDIA. All software tools were run on Ubuntu 16.04 with 32 CPU cores and 256 GB memory (without setting memory limitations in the benchmarked software tools). The data were processed twice by DreamDIA with different numbers of acquisition cycles specified for each precursor to analyze, and the time consumptions were both compared.

Supplementary Tables

Supplementary Table 1. Identification performance evaluation of DreamDIA when different hyper-parameters are used for the deep representation models. We considered 48 combinations of different numbers of neurons for the layers in the neural network. For each hyperparameter combination, the model was built and trained on the same training set with best epoch selected when the validation loss stopped decreasing after at least 10 epochs. Then each model was used by DreamDIA to analyze the S1-1 run of the MCB dataset with the two-species library, and the number of mouse precursors identified at 1% proxy FDR was compared.

N neurons LSTM1	N neurons LSTM2	N neurons FC1	N identified at 1% FDR
128	64	32	62676
128	128	16	62598
128	64	4	62565
128	128	64	62539
128	128	32	62512
128	16	4	62500
64	64	64	62485
128	8	8	62470
128	64	16	62448
64	32	4	62448
128	64	8	62443
128	32	8	62443
64	64	4	62437
128	128	128	62427
128	32	32	62427
64	32	32	62422
128	4	4	62399
128	32	16	62382
64	16	4	62379
128	8	4	62376
64	16	8	62374
128	128	8	62372
64	8	8	62368
64	32	8	62353
64	64	8	62344
64	64	32	62331
32	16	16	62331
128	16	16	62326
32	16	4	62318
128	128	4	62318

128	64	64	62314
128	32	4	62309
128	16	8	62299
64	16	16	62282
64	32	16	62242
64	8	4	62242
32	32	4	62231
32	32	8	62219
64	64	16	62200
32	8	4	62185
32	16	8	62180
32	32	32	62141
16	16	4	62104
16	16	8	62094
32	32	16	62080
32	8	8	62070
8	8	4	61969
8	4	4	61622

Supplementary Table 2. LFQbench test results on the HYE110 dataset. The global accuracy metric provided by LFQbench software suite reflects the median deviation of calculated log-ratios to the expected value. The absolute value of the global accuracy was used for comparison.

	**human peptide global accuracy**	**human protein global accuracy**	**yeast peptide global accuracy**	**yeast protein global accuracy**	***E.coli* peptide global accuracy**	***E.coli* protein global accuracy**
DreamDIA	0	0	0.0505	0.1269	0.1007	0.0079
OpenSWATH	0	0	0.7268	0.5604	0.7429	0.6205
DIA-NN	0	0	0.2623	0.1256	0.3132	0.2124

Supplementary Table 3. LFQbench test results on the HYE124 dataset. The global accuracy metric provided by LFQbench software suite reflects the median deviation of calculated log-ratios to the expected value. The absolute value of the global accuracy was used for comparison.

	**human peptide global accuracy**	**human protein global accuracy**	**yeast peptide global accuracy**	**yeast protein global accuracy**	***E.coli* peptide global accuracy**	***E.coli* protein global accuracy**
DreamDIA	0	0	0.0009	0.0038	0.1950	0.1343
OpenSWATH	0	0	0.2315	0.1590	0.5198	0.3954
DIA-NN	0	0	0.0100	0.0030	0.3073	0.2557

Supplementary Table 4. LFQbench test results of DreaDIA on the HYE110 dataset with different fragment ions for quantification. The global accuracy metric provided by LFQbench software suite reflects the median deviation of calculated log-ratios to the expected value. The absolute value of the global accuracy was used for comparison.

**fragments for quantification**	**human peptide global accuracy**	**human protein global accuracy**	**yeast peptide global accuracy**	**yeast protein global accuracy**	***E.coli* peptide global accuracy**	***E.coli* protein global accuracy**
library	0	0	0.0505	0.1269	0.1007	0.0079
library + top 3 self	0	0	0.0892	0.1372	0.0548	0.0280
library + top 6 self	0	0	0.0438	0.0965	0.0840	0.0099
library + top 9 self	0	0	0.0123	0.0845	0.1313	0.0289
library + top 12 self	0	0	0.0154	0.0808	0.1293	0.0383
library + top 15 self	0	0	0.0079	0.0685	0.1404	0.0588

Supplementary Table 5. Datasets used in this work.

Dataset	N runs used	Equipment	Year	Dataset ID	application
L929 mouse dataset	3	TripleTOF 5600	2020	PXD021390	Training
HEK293 dataset	3	Orbitrap Fusion Lumos	2020	PXD015098	Training
BiolDS-OT dataset	4	Q Exactive HF-X	2020	PXD016647	Training
Mouse cerebellum dataset	10	Orbitrap Fusion Lumos	2020	PXD011691	Testing
LFQbench 64var TTOF6600 dataset	12	TripleTOF 6600	2018	PXD002952	Testing
HeLa dataset	4	Q Exactive HF	2017	PXD005573	Testing

Supplementary Notes

1.Identification of more deamidated peptides with DreamDIA

The identification of post-translational modification (PTM) peptides is both crucial and challenging for peptide-centric scoring (PCS) softwares. The MS2 spectra originated from related peptides including the non-modified peptide, or modified peptides with the same sequence and the same modifications at different amino acid sites, or peptides with the same sequence and isobaric modifications can be highly similar [1]. Among all the known PTMs, deamidation is one of the most difficult modifications for PCS sotware tools to accurately identify due to its extremely small mass shift of 0.9840 Da. Herein, we compared the ability of DreamDIA to identify deamidated peptides with that of DIA-NN using the pseudo-modification method proposed by the authors of DIA-NN. More specifically, the test data were analyzed twice by each software tool, using the library containing common deamidated peptides with mass shift of 0.9840 Da for the first time, and of 1.0227 Da for the second time. The difference between these two mass shifts is exactly two-fold of the mass difference between ^{13}C isotope (1.0034 Da) and the deamidation modification mass shift (0.9840 Da). The PCS software should identify more deamidated peptides in the first analysis and fewer in the second analysis. With FDR calculated by the two-species method, DreamDIA identified nearly 1.5-fold more deamidated peptides compared with DIA-NN in the first analysis, and slightly fewer deamidated peptides in the second analysis (Supplementary Figure 4).

2. Auxiliary scores in DreamDIA

In addition to the deep representation features output from the deep representation model, we included several auxiliary scores for candidate peak groups of each precursor in DreamDIA, as listed below.

(1) Difference between the real RT and the RT recorded in the library.
(2) Square of the difference between the real RT and the RT recorded in the library.
(3) Cosine similarity of the real intensities and library intensities of all the fragments.
(4) Mean and standard deviation of the three scores above of all the candidate peak groups for each precursor.
(5) Length of the peptide sequence.
(6) Charge of the precursor.
(7) m/z of the precursor.

3. Skyline step-by-step settings

We analyzed the MCB dataset by Skyline [2] for the performance benchmarking of DreamDIA. We followed most of the settings provided by LFQbench [3], and the detailed procedures were shown below.

(1) Open Skyline.
(2) Blank Document.
(3) Settings -> Transition Settings:
    Full-Scan:
        Acquisition method: DIA;
        Product mass analyzer: Orbitrap;
        Isolation scheme: input the isolation window settings manually;
        Resolving power: At: 60,000. 400 m/z;
        Retention time filtering: Use only scans within 10;
    Instrument:
        Min m/z: 50 m/z;
        Max m/z: 2000 m/z;
        Method match tolerance m/z: 0.01 m/z;
    Library:
        Ion match tolerance: 0.5 m/z;
        If a library spectrum is available, pick its most intense ions: checked;
        Pick: 6 product ions
        From filtered ion charges and types;
    Filter:
        Precursor charges: 2, 3, 4, 5;
        Ion charges: 1, 2;
        Ion types: y, b;
        Product ion selection:
            From “ion 3”;
            To “last ion - 1”;
            Special ions:
                N-terminal to Proline: checked;
        Use DIA precursor window for exclusion: checked;
        Auto-select all matching transitions: checked;
(4) Settings -> Peptide Settings:
    Modifications:
        Structural modifications:
            Gln -> pyro-Glu (N-term Q): “Variable” checked;
            Pyro-carbamidomethyl (N-term C): “Variable” checked;
            Oxidation (M): “Variable” checked;
            Glu -> pyro-Glu (N-term E): “Variable” checked;
            Carbamyl (N-term) without H: “Variable” checked;
            Carbamidomethyl (C): “Variable” unchecked;
        Max varable mods: 3;
        Max neural losses: 1;
        Isotope label type: heavy;
        Isotope modifications:
            Label: 13C(6)15N(2) (C-term K) checked;
            Label: 13C(6)15N(4) (C-term R) checked;

Internal standard type: light;

Filter:

Min length: 7;

Max length: 36;

Exclude N-terminal AAs: 36;

Auto-select all matching peptides: checked;

Prediction:

Use measured retention times when present: checked;

Time window: 2min;

(5) File -> Import -> transition List.

Skip the warning window;

Skip the iRT calculator building window;

Create library;

(6) Settings -> Peptide Settings:

Prediction:

Retention time predictor: CiRT (iRT-C18);

Add the library;

Maximum transitions per peptide: 6.

(7) Refine -> Add Decoys.

Decoy generation method: Reverse Sequence;

(8) Settings -> Integrate All.

(9) Save the document.

(10) File -> Import -> Results -> Add single-injection replicates in files -> OK.

(11) Refine -> Reintegrate:

Peak scoring model: Add;

Choose model: mProphet;

Use decoys: checked;

Check all of the available feature scores;

Train;

OK;

Integrate all peaks;

Overwrite manual integration: checked;

OK;

(12) File -> Export -> Report.

The report template (SWATHbenchmark_long.skr) provided by LFQbench [3] was used.

(13) Options that are not mentioned above were ignored and their default settings were used.

Supplementary References

[1] Christina, L. *et al.* Data-independent acquisition-based SWATH-MS for quantitative proteomics: a tutorial. *Molecular Systems Biology* **14**(8), e8126 (2018).

[2] MacLean, B. *et al.* Skyline: an open source document editor for creating and analyzing targeted proteomics experiments. *Bioinformatics* **26**, 966-968 (2010).

[3] Navarro, P. *et al.* A multicenter study benchmarks software tools for label-free proteome quantification. *Nature Biotechnology* **34**, 1130-1136 (2016).
