# Supplementary material for: Deep representation features from DreamDIAXMBD improve the analysis of data-independent acquisition proteomics
Source: Commun Biol. 2021 Oct 14;4:1190. doi: 10.1038/s42003-021-02726-6 (PMC8517002; doi:10.1038/s42003-021-02726-6)
Supplement: Supplementary file 3 — Description of Additional Supplementary Files [file 42003_2021_2726_MOESM3_ESM.pdf]

Description of Additional Supplementary Files

File name: Supplementary Data 1

Description: Source data for Figure 2a.

File name: Supplementary Data 2

Description: Source data for Figure 2b.

File name: Supplementary Data 3

Description: Source data for Figure 3.

File name: Supplementary Data 4

Description: Source data for Figure 4a.

File name: Supplementary Data 5

Description: Source data for Figure 4b.
