# Supplementary material for: Deep representation features from DreamDIAXMBD improve the analysis of data-independent acquisition proteomics
Source: Commun Biol. 2021 Oct 14;4:1190. doi: 10.1038/s42003-021-02726-6 (PMC8517002; doi:10.1038/s42003-021-02726-6)
Supplement: Supplementary file 9 — Reporting Summary [file 42003_2021_2726_MOESM9_ESM.pdf]

nature portfolio

Corresponding author(s): Rongshan Yu and Jiahuai Han

Last updated by author(s): Sep 9, 2021

Reporting Summary

Nature Portfolio wishes to improve the reproducibility of the work that we publish. This form provides structure for consistency and transparency in reporting. For further information on Nature Portfolio policies, see our Editorial Policies and the Editorial Policy Checklist.

Statistics

For all statistical analyses, confirm that the following items are present in the figure legend, table legend, main text, or Methods section.

n/a	Confirmed	
☐	☒	The exact sample size (*n*) for each experimental group/condition, given as a discrete number and unit of measurement
☐	☒	A statement on whether measurements were taken from distinct samples or whether the same sample was measured repeatedly
☐	☒	The statistical test(s) used AND whether they are one- or two-sided<br>*Only common tests should be described solely by name; describe more complex techniques in the Methods section.*
☒	☐	A description of all covariates tested
☐	☒	A description of any assumptions or corrections, such as tests of normality and adjustment for multiple comparisons
☐	☒	A full description of the statistical parameters including central tendency (e.g. means) or other basic estimates (e.g. regression coefficient) AND variation (e.g. standard deviation) or associated estimates of uncertainty (e.g. confidence intervals)
☒	☐	For null hypothesis testing, the test statistic (e.g. *F*, *t*, *r*) with confidence intervals, effect sizes, degrees of freedom and *P* value noted<br>*Give P values as exact values whenever suitable.*
☒	☐	For Bayesian analysis, information on the choice of priors and Markov chain Monte Carlo settings
☐	☒	For hierarchical and complex designs, identification of the appropriate level for tests and full reporting of outcomes
☒	☐	Estimates of effect sizes (e.g. Cohen's *d*, Pearson's *r*), indicating how they were calculated

Our web collection on statistics for biologists contains articles on many of the points above.

Software and code

Policy information about availability of computer code

Data collection	Publicly available data were used for the training of deep representation models and the evaluation of DIA analysis tools.
Data analysis	ProteoWizard (v3.0.19317) was used to transform the raw MS data files to the .mzML or .mzXML formats. For DreamDIA (v2.0.2), centroided .mzXML files were used as input. For Skyline-daily (v21.0.9.118) and DIA-NN (v1.7.11), centroided .mzML files were used. For OpenSWATH (v2.6.0), profile .mzXML files were used as recommended. The output of OpenSWATH was further processed by Pyprophet-cli (v0.0.19). Statistical analysis was performed in the R environment (v3.6.3) and the Python environment (v3.6.10).

For manuscripts utilizing custom algorithms or software that are central to the research but not yet described in published literature, software must be made available to editors and reviewers. We strongly encourage code deposition in a community repository (e.g. GitHub). See the Nature Portfolio guidelines for submitting code & software for further information.

Data

Policy information about availability of data

All manuscripts must include a data availability statement. This statement should provide the following information, where applicable:

- Accession codes, unique identifiers, or web links for publicly available datasets
- A description of any restrictions on data availability
- For clinical datasets or third party data, please ensure that the statement adheres to our policy

The datasets used in this study are publicly available at the ProteomeXchange Consortium (http://proteomecentral.proteomexchange.org/) via the PRIDE or iProX partner repository. The dataset identifiers are PXD015098, PXD021390, PXD011691, PXD005573, PXD016647 and PXD002952.

Field-specific reporting

Please select the one below that is the best fit for your research. If you are not sure, read the appropriate sections before making your selection.

☒ Life sciences ☐ Behavioural & social sciences ☐ Ecological, evolutionary & environmental sciences

For a reference copy of the document with all sections, see nature.com/documents/nr-reporting-summary-flat.pdf

Life sciences study design

All studies must disclose on these points even when the disclosure is negative.

Sample size	In this study, the numbers of precursors, peptides and proteins identified and quantified by different software tools were evaluated. The sample sizes were determined by the sizes of the public datasets themselves, including n = 3 for the LFQbench dataset, n = 4 for the HeLa dataset and up to n = 10 for the MC dataset. In all cases, the sample sizes were considered sufficient in view of the effect magnitudes observed in comparison to the low technical variability.
Data exclusions	No data were excluded from the analyses.
Replication	All attempts at replication were successful.
Randomization	Randomization is not relevant for this study, since it presents computational benchmarks, wherein different software tools were used to analyze the same data.
Blinding	Blinding was not considered necessary, as this work does not involve any experiments and data processing was performed in an unbiased automatic fashion.

Reporting for specific materials, systems and methods

We require information from authors about some types of materials, experimental systems and methods used in many studies. Here, indicate whether each material, system or method listed is relevant to your study. If you are not sure if a list item applies to your research, read the appropriate section before selecting a response.

Materials & experimental systems

n/a	Involved in the study
☒	☐ Antibodies
☒	☐ Eukaryotic cell lines
☒	☐ Palaeontology and archaeology
☒	☐ Animals and other organisms
☒	☐ Human research participants
☒	☐ Clinical data
☒	☐ Dual use research of concern

Methods

n/a	Involved in the study
☒	☐ ChIP-seq
☒	☐ Flow cytometry
☒	☐ MRI-based neuroimaging
